# Reporting LDL cholesterol results by clinical biochemistry laboratories in Czechia and Slovakia to improve the detection rate of familial hypercholesterolemia

**DOI:** 10.11613/BM.2023.030705

**Published:** 2023-10-15

**Authors:** Tomáš Šálek, Vladimír Soška, Marek Budina, Marek Vecka, Veronika Šálková, Michal Vrablík

**Affiliations:** 1 Institute of Laboratory Medicine, Medical Faculty, University of Ostrava, Ostrava, Czechia; 2 Department of Clinical Biochemistry and Pharmacology, The Tomas Bata Hospital in Zlín, Zlín, Czechia; 3 Department of Clinical Biochemistry, St. *Anne’s* University Hospital Brno, Brno, Czechia; 4 Second Clinic of Internal Medicine, Faculty of Medicine, Masaryk University Brno, Czechia; 5 SEKK, spol. s. r. o., Pardubice, Czechia; 6 Institute of Medical Biochemistry and Laboratory Diagnostics, Faculty General Hospital and First Faculty of Medicine, Charles University, Prague, Czechia; 7 Third Department of Medicine, General University Hospital and First Faculty of Medicine, Charles University, Prague, Czechia

**Keywords:** familial hypercholesterolemia, technical report, HDL cholesterol, LDL cholesterol, apolipoprotein B, lipoprotein(a)

## Abstract

**Introduction:**

This survey aims to assess the implementation of recommendations from the European Atherosclerosis Society (EAS) and the European Federation of Clinical Chemistry and Laboratory Medicine (EFLM) by clinical biochemistry laboratories in Czechia and Slovakia in their policies for reporting low-density lipoprotein cholesterol (LDL-C) concentrations.

**Materials and methods:**

The web-based survey was distributed to all 383 Czech and Slovak clinical biochemistry laboratories that measure lipids by external quality assessment provider SEKK. A total of 17 single-answer questions were included. The questionnaire was focused on the detection and decision points in familial hypercholesterolemia (FH). All survey answers were taken into account. The laboratories followed the EFLM and EAS guidelines when they reported an interpretative comment considering FH diagnosis in adults.

**Results:**

A total of 203 (53%) laboratories answered. Only 5% of laboratories added interpretative comments considering FH diagnosis when LDL-C concentrations are above 5.0 mmol/L in adults, and 3% of laboratories added interpretative comments considering FH diagnosis when LDL-C concentrations are above 4.0 mmol/L in children. Only 7% of laboratories reported goals for all cardiovascular risk categories (low, moderate, high, very high). Non-HDL cholesterol concentrations were calculated by 74% of responders. A significant number (51%) of participants did not measure apolipoprotein B, and 59% of laboratories did not measure lipoprotein(a).

**Conclusions:**

Only a small portion of laboratories from Czechia and Slovakia reported high LDL-C results with interpretative comments considering FH diagnosis in adults, the laboratories did not follow the guidelines.

## Introduction

Familial hypercholesterolemia (FH) is the most common monogenic disorder with autosomal dominant inheritance. The global prevalence rate of FH is approximately 1:300 (*1*). Children who have one parent with a heterozygous form have a 50% probability of having the disease. It is characterized by high concentrations of low-density lipoprotein cholesterol (LDL-C) (*2*). This condition is clinically characterized by premature atherosclerotic cardiovascular disease (ASCVD). The basic lipid profile includes total cholesterol, triacylglycerols, LDL-C, and high-density lipoprotein cholesterol (HDL-C). The typical laboratory feature of FH is serum LDL-C concentrations above 5 mmol/L in adult patients and LDL-C concentrations above 4 mmol/L in children. The disease’s course can be asymptomatic; the first symptom may be a major cardiovascular event or sudden death. Current effective treatment of FH includes mainly non-pharmacological treatment (physical activity with a healthy diet), usually accompanied by pharmacotherapy that consists of statins, ezetimibe, PCSK-9 inhibitors, small interfering RNA molecules, and lipoprotein apheresis because, in most cases, non-pharmacological measures do not get LDL-C at a healthy level (*3*). The “Make Early Diagnosis and Prevent Early Death” (MED-PED) project is a worldwide activity to detect this condition. The Czech Republic is an active participant in this international venture, with a current FH detection rate of approximately 20% in the national register (*4*, *5*). Slovak colleagues also participate in the MED-PED project. Unfortunately, the majority of affected FH patients in the world are not diagnosed (*6*). Alert letters in patients with high LDL-C concentrations improve the FH detection rate (*7*). Clinical biochemistry professionals may improve the FH detection rate by adding interpretative comments to serum LDL-C concentrations above 5 mmol/L in adult patients and serum LDL cholesterol concentrations above 4 mmol/L in children. The comment “Consider heterozygous FH.” should be provided in the laboratory report. This interpretative comment is recommended by both the European Federation of Clinical Chemistry and Laboratory Medicine (EFLM) and the European Atherosclerosis Society (EAS) (*8*). The evidence supporting LDL-C thresholds of 5 mmol/L in adults and 4 mmol/L in children is derived from LDL-C concentrations of first-degree relatives of genetically confirmed FH patients (*9*). The importance of FH detection was recognized at the international level, and the Prague declaration for FH screening across Europe was signed by European Union representatives. The screening approach is cost-effective (*10*). Implementation of the guidelines was not suggested to laboratories prior to this study.

The low FH detection rate is the primary reason we performed the questionnaire study, which aims to assess the implementation of recommendations from EAS and EFLM by clinical biochemistry laboratories in Czechia and Slovakia in their policies on reporting LDL-C concentrations (*6*).

## Materials and methods

The web-based survey was distributed to all 383 Czech and Slovak clinical laboratories that measure lipids in external quality assessment (EQA) provided by SEKK (*11*). There were 31 laboratories from Slovakia and 172 laboratories from Czechia. Since Slovakia does not have its own EQA, Slovakia laboratories participate in Czechia cycles and follow similar policies. The study was performed in January 2023 in a regular EQA scheme. The questionnaire was created by three experts from the Czech Society for Atherosclerosis based on recommendations from EFLM and EAS. The most important questions focused on the detection and decision points in FH. Another three laboratory professionals from the EQA organization validated the content. The laboratories followed the EFLM and EAS guidelines when they reported an interpretative comment considering FH diagnosis in adults.

Laboratories received a notification e-mail before the survey. The survey was entered and distributed by the SEKK website to all participants. The answers were also entered electronically at the website, together with the lipid measurement results. Participation in the study was voluntary. No surveys were excluded.

All single-answer questions and possible responses are shown in Table 1. Laboratories were informed that their answers would be anonymously published. The survey was also intended to further educate laboratory professionals. Laboratories received recommendations after the completion of the study.

**Table 1:** Questionnaire and results from the survey on adherence to EAS and EFLM guidelines on FH among Czech and Slovak laboratories

**Question**	**Possible answers**	**N (%)**
1. What is your laboratory type?	Hospital	73 (36)
	Laboratory for outpatients clinics	126 (62)
	Without answer	4 (2)
2. How do you report LDL-C results?	We only measure LDL-C	117 (58)
	We calculate LDL-C	43 (21)
	We measure and calculate LDL-C	35 (17)
3. If you calculate LDL-C, what equation do you use?	Friedewald	84 (41)
	Martin – Hopkins	1 (1)
	Another equation	0
4. When do you report calculated LDL-C?	In all cases	4 (2)
	Requirements for triacylglycerols and/or LDL-C concentrations should be met	74 (37)
	Only when the physician requests	10 (5)
5. When do you report measured LDL-C?	In all cases	52 (26)
	Requirements for triacylglycerols concentration should be met	78 (38)
6. Do you report an interpretative comment when LDL-C concentration is above 5 mmol/L in adults?	No	156 (77)
	No, but we plan to start it this year	23 (11)
	Yes	10 (5)
7. Do you report an interpretative comment when children’s LDL-C concentration is above 4 mmol/L?	No	158 (78)
	No, but we plan to start it this year	23 (11)
	Yes	7 (3)
8. Interpretative comments to high LDL-C concentrations	We do not report	148 (73)
	It is written automatically by LIS	9 (4)
	We select one option from LIS	0
	An individual comment is written manually	23 (11)
9. Do you mention the possible diagnosis of Familial hypercholesterolemia when you report an interpretative comment to a high LDL-C concentration?	Yes	11 (5)
	No	82 (40)
10. Do you mention the local Familial hypercholesterolemia center when you report an interpretative comment to a high LDL-C concentration?	Yes	6 (3)
	No	88(43)
11. What is your LDL-C lower reference limit on the laboratory report?	Less than 0.5 mmol/L	25 (12)
	0.5 mmol/L	9 (4)
	1.0 mmol/L	13 (6)
	Higher than 1.0 mmol/L	129 (64)
12. What is your LDL-C upper reference limit on the laboratory report?	1.0 mmol/L	0
	1.4 mmol/L	0
	1.8 mmol/L	0
	2.6 mmol/L	10 (5)
	3.0 mmol/L	171 (84)
13. What is the target LDL-C concentration in a patient at very high cardiovascular risk after one heart attack?	< 1.0 mmol/L	9 (4)
	< 1.4 mmol/L	69 (34)
	< 1.8 mmol/L	31 (15)
	< 2.6 mmol/L	24 (12)
	< 3.0 mmol/L	12 (6)
14. Do you report on your laboratory report LDL-C goals for all cardiovascular risk categories? (low, moderate, high, very high)	Yes	15 (7)
	No	152 (75)
	No, but we plan to start it this year	16 (8)
15. Do you report non-HDL-C concentrations?	No, but we plan to start it this year	40 (20)
	Yes, on request	17 (8)
	Yes, automatically, when total and HDL-C concentrations are available	133 (66)
16. Does your laboratory measure apolipoprotein-B concentrations?	No	99 (49)
	No, but we plan to start it this year	5 (2)
	Yes	89 (44)
17. Does your laboratory measure lipoprotein(a) concentrations?	No	113 (56)
	No, but we plan to start it this year	6 (3)
	Yes, in molar units	43 (21)
	Yes, in mass units	30 (15)
The denominator for all percentage calculations was defined as the number of all participating laboratories (N = 203). LDL-C - low density lipoprotein cholesterol. LIS - laboratory information system. non-HDL-C – non high density lipoprotein cholesterol.		

### Statistical analysis

The Microsoft Office Excel program (Microsoft, Washington, USA) was used for data processing. The denominator for all percentage calculations was 203. The differences were assessed by Chi-square test using MedCalc 20.121 statistical software (MedCalc, Ostend, Belgium). The level of significance was defined as P < 0.05.

## Results

A total of 203 laboratories participated in the study. The response rate was 53%, with 172 of the laboratories being from Czechia and 31 from Slovakia. Only 22 laboratories answered all questions. Only 5% of laboratories followed the guidelines by adding interpretative comments considering FH diagnosis when LDL-C concentrations are above 5.0 mmol/L in adults. Responses to the questions are displayed in Table 1.

A high portion of participants (51%) did not measure apolipoprotein B, and 59% of laboratories did not measure lipoprotein (a) (Lp(a)).

Czech laboratories reported more frequently non-HDL-C (P = 0.010) and more frequently measured Lp(a) in molar units (P = 0.030). Slovak laboratories reported more frequently lower LDL-C limit of ≤ 0.5 mmol/L (P = 0.012), upper LDL-C limit of 2.6 mmol/L (P = 0.001), and goals for all cardiovascular risk categories (P = 0.044).

The differences in answers between hospital and non-hospital laboratories are displayed in Table 2.

**Table 2:** The differences in answers between hospital and non-hospital laboratories

**Answer to question by the laboratory**	**Hospital labs** **(N = 73)**	**Non-hospital labs** **(N = 126)**	**P**
Only measures LDL-C, N(%)	36 (49)	78 (62)	0.084
Uses Friedewald equation, N (%)	37 (51)	47 (37)	0.066
Mentions possible diagnosis of FH in selected cases, N (%)	6 (8)	5 (4)	0.207
Lower LDL-C limit ≤ 0.5 mmol/L, N (%)	9 (12)	24 (19)	0.221
Upper LDL-C limit 2.6 mmol/L, N (%)	3 (4)	7 (6)	0.628
Reports goals for all cardiovascular risk categories, N(%)	2 (3)	13 (10)	0.052
Reports non-HDL-C, N (%)	47 (64)	84 (67)	0.744
Measures apolipoprotein B, N (%)	30 (41)	56 (44)	0.647
Measures lipoprotein(a) in nmol/L units, N(%)	17 (23)	26 (21)	0.662
LDL-C - low density lipoprotein cholesterol. FH - familial hypercholesterolemia. non-HDL-C – non high density lipoprotein cholesterol. P value < 0.05 was considered statistically significant.			

## Discussion

We found that only a small portion of laboratories from Czechia and Slovakia reported high LDL-C results with interpretative comments that considered an FH diagnosis.

De Wolf *et al.* showed that 83% of laboratories did not add alert comments when FH diagnosis was suspected (*12*). We found similarly unsatisfactory results. It is recommended that laboratories add an alert to possible life-threatening results. Notification of possible FH diagnosis may increase the final detection rate of this condition. Automated electronic commenting may prevent overlooking and transcription errors.

Silva *et al.* reported the need for standardization of laboratory reports with the inclusion of target goals for different cardiovascular risk (*13*). Only a few laboratories in our study reported treatment targets for different risk categories. The upper and lower limits are reported with most tests on every laboratory report. Limits may be derived from the highest and lowest treatment goals. The 2021 ESC Guidelines on cardiovascular disease prevention in clinical practice recommended an upper LDL-C target of 2.6 mmol/L. The lower limit of 0.5 mmol/L is 50% of the goal of 1.0 mmol/L for patients who experienced two cardiovascular events within the last two years (*14*-*16*). However, recent studies have shown that even lower LDL-C concentrations are both beneficial and safe (*17*, *18*). Some physicians could discontinue treatment when the LDL-C results go below the lower limit. In order to assure safe treatment, it is very important for LDL-C results to remain within decision thresholds. Laboratory professionals should be aware of low safe LDL-C concentrations and set their decision limits based on the latest studies. The differences among LDL-C measurement and calculation methods are not reflected in the guidelines.

Low-density lipoprotein cholesterol was calculated by the Friedewald equation in most studies (*19*). This formula cannot be used when triacylglycerol concentrations are above 4.5 mmol/L, as it underestimates LDL-C concentrations in the range below 1.8 mmol/L (*20*). This was the reason that alternative equations (*e.g.,* Martin-Hopkins) were developed so as to provide a more accurate measure for risk classification than the Friedewald equation (*21*). This modified equation may be preferable for the calculation of LDL-C, particularly for patients with low LDL-C concentrations < 1.8 mmol/L and/or triacylglycerol concentrations 2.0 - 4.5 mmol/L and in non-fasting samples (*22*). Currently, laboratories can measure LDL-C directly, but it is important to note that direct LDL-C measurements also have limitations, including systematic bias and inaccuracy in patients with dyslipidaemia, especially for high triacylglycerol concentrations (*23*). Directly measured LDL-C agrees with the reference beta-quantification method only in healthy subjects but exhibits positive bias for subjects with hypertriglyceridemia in diseased subjects (*24*). In our study, the majority of laboratories only measured LDL-C on automated clinical biochemistry instruments.

The measurement LDL-C alone does not reflect all atherogenic lipid particles in the blood. Calculation of non-HDL cholesterol and Systemic Coronary Risk Estimation 2 (SCORE2) were developed for better identification of patients at residual cardiovascular risk (*25*). The 2021 ESC Guidelines on cardiovascular disease prevention in clinical practice assign cardiovascular risk according to non–HDL cholesterol concentrations (*14*). The majority of laboratories in Czechia and Slovakia calculated and reported non-HDL cholesterol concentrations.

In 13,015 statin-treated patients from the Copenhagen General Population Study, serum apolipoprotein–B concentration was shown to be a more accurate marker of both all-cause mortality and myocardial infarction risk than non-HDL cholesterol or LDL-C (*26*). This ability to identify patients at ASCVD risk is the reason for the implementation of apolipoprotein B more frequently in routine clinical practice.

Lipoprotein (a) is another causal risk factor for the development of atherosclerosis, and it should be measured at least once during a person’s lifetime. People who have Lp(a) higher than 430 nmol/L have similar ASCVD risk as people who are heterozygous for FH. A 221 nmol/L change in plasma Lp(a) concentrations was associated with the same coronary heart disease risk as a 1 mmol/L change in LDL-C concentrations (*27*). This is the reason all people should undergo Lp(a) measurement at least once in their lifetime. A total of 36% of clinical biochemistry laboratories in Czechia and Slovakia measured Lp(a) concentrations.

Molar units are currently recommended for Lp(a) measurement due to the size heterogeneity of this molecule. The primary reference material of Lp(a) was developed in molar units (*28*). In our study, 59% of laboratories that measure Lp(a) reported results in nmol/L units.

The recommendations were published in the national bulletin Fons to all laboratories after the survey was completed (*29*).

Laboratories were recommended to:

Report interpretative comments considering FH diagnosis in adult patients with LDL-C concentrations above five mmol/L and children with LDL-C concentrations above four mmol/L.
Provide information on target LDL-C concentrations for all cardiovascular risk categories in the laboratory report.
Provide the upper decision threshold value in the laboratory report for the population at low risk (2.6 mmol/L), and the lower decision threshold value in the laboratory report should be the goal for the population at highest risk (0.5 mmol/L).
Not to use the Friedewald equation when plasma triacylglycerol concentrations exceed 4.5 mmol/L.
Use the Martin-Hopkins equation in patients with LDL-C concentrations below 1.8 mmol/L, in triacylglycerol concentration ranges of 2.0 – 4.5 mmol/L, and in non-fasting samples.
Calculate non-HDL cholesterol concentrations.
Start measuring apolipoprotein B
Start measuring Lp(a) in molar units.

The Czech Society for Clinical Biochemistry and the Czech Society for Atherosclerosis published a statement for diagnosing dyslipidaemias in 2017 (*30*). The poor implementation of current international EAS/EFLM recommendations may be caused by the fact that recommendations are in English language. Of course, the interpretation of the LDL-C results might be influenced by the presence of other diseases like diabetes, hypothyroidism, nephrotic syndrome, and some hepatic diseases. The first step in the diagnosis of primary hypercholesterolemia is the exclusion of secondary hypercholesterolemia. An interpretation comment could recommend glucose, thyroid-stimulating hormone, liver function tests, and proteinuria testing. The limitations of this study are an incomplete response rate, as some laboratories did not answer all questions, and an inability to verify their answers. Data on LDL-C calculation are not concordant. A total of 85 participants provided information on the equation, but only 78 laboratories reported that they calculated LDL-C. Some respondents probably misunderstood the questions.

We conclude that the laboratories did not follow the guidelines, because only a small portion of laboratories from Czechia and Slovakia report high LDL cholesterol results with interpretative comments considering FH diagnosis in adults. More laboratories should consider apolipoprotein-B and Lp(a) measurements to refine the estimation of cardiovascular risk. We believe in the educational benefits of both the questionnaire used and this article.

## Data Availability

All data generated and analysed in the presented study are included in this article.
